# Prevalence and Diagnostic Challenges of Cytomegalovirus Colitis in Inflammatory Bowel Disease: A Retrospective Study From a Romanian Tertiary Care Center

**DOI:** 10.7759/cureus.102764

**Published:** 2026-02-01

**Authors:** Sergiu Frandes, Oana Frandes, Melania Macarie, Simona M Bataga

**Affiliations:** 1 Gastroenterology, George Emil Palade University of Medicine, Pharmacy, Science, and Technology of Târgu Mureș, Târgu Mureș, ROU; 2 Anesthesia and Critical Care, George Emil Palade University of Medicine, Pharmacy, Science, and Technology of Târgu Mureș, Târgu Mureș, ROU; 3 Internal Medicine, George Emil Palade University of Medicine, Pharmacy, Science, and Technology of Târgu Mureș, Târgu Mureș, ROU

**Keywords:** crohn's disease, cytomegalovirus, immunohistochemistry, inflammatory bowel disease, serology, ulcerative colitis

## Abstract

Background and aims: Cytomegalovirus (CMV) colitis is a clinically significant complication in patients with inflammatory bowel disease (IBD), particularly in those with severe or steroid-refractory disease, where it may contribute to adverse outcomes. This study aimed to determine the frequency of acute CMV infection and tissue-confirmed CMV colitis among hospitalized IBD patients who underwent CMV evaluation.

Methods: We conducted a retrospective study including adult patients with IBD hospitalized between April 1, 2024, and December 31, 2024, at the Emergency Clinical County Hospital of Târgu Mureș, Romania. CMV serology (immunoglobulin G (IgG), immunoglobulin M (IgM)), colonoscopy with biopsy, and immunohistochemistry were performed when clinically indicated. Demographic characteristics, clinical presentation, treatment regimens, laboratory parameters, and histopathologic findings were analyzed for all included patients.

Results: 54 IBD patients were evaluated. The mean age was 41 years, and most were male. Crohn's disease was diagnosed in 24 patients (44.44%) and ulcerative colitis in 30 patients (55.56%). Three patients (5.55%) showed evidence of acute CMV infection based on serology. Only one patient (1.85%) was diagnosed with CMV colitis by immunohistochemistry. All patients with evidence of CMV infection had received corticosteroid therapy, although the small number of cases limits interpretation.

Conclusions: CMV colitis was uncommon in this cohort of evaluated patients. Diagnosis primarily relied on histopathologic confirmation, with serology serving only as a limited adjunct. CMV infection should be considered in patients with active or steroid-refractory disease. Observations regarding corticosteroid exposure should be interpreted cautiously and considered hypothesis-generating.

## Introduction

Inflammatory bowel diseases (IBD), including Crohn's disease (CD) and ulcerative colitis (UC), are chronic, relapsing inflammatory conditions of the gastrointestinal tract characterized by alternating periods of exacerbation and remission. CD may affect any segment of the gastrointestinal tract, most commonly the terminal ileum and colon, and is defined histologically by transmural inflammation, fissuring ulcers, and sometimes non-caseating granulomas. In contrast, UC is limited to the colonic mucosa and typically progresses proximally from the rectum in a continuous pattern. The global prevalence of IBD has risen significantly in recent decades, reflecting changes in environmental exposures and lifestyle factors [1-4].

Patients with IBD are at increased risk for viral and bacterial infections, including cytomegalovirus (CMV), due to immune dysregulation, epithelial barrier dysfunction, malnutrition, and the use of immunosuppressive therapies such as corticosteroids or azathioprine [5]. CMV is a ubiquitous β-herpesvirus that establishes lifelong latency after primary infection and may reactivate in states of impaired cellular immunity, including severe or steroid-refractory IBD [6-9]. Previous studies have reported CMV reactivation rates ranging from 21% to 34% in moderate-to-severe colitis and exceeding 30% in steroid-refractory disease [7,9]. CMV colitis has been associated with poor outcomes, including toxic megacolon, increased hospitalization, and a higher risk of colectomy [1].

Diagnosing CMV colitis remains challenging. Although several diagnostic modalities exist, including serology, antigenemia assays, quantitative polymerase chain reaction (PCR), histopathology, and immunohistochemistry (IHC), their sensitivity and specificity vary considerably [10]. Serology has limited diagnostic value due to high immunoglobulin G (IgG) seroprevalence in the general population and may be confounded by immunosuppression [11]. Endoscopic features are nonspecific and may include erythema, erosions, ulcerations, or pseudotumor-like lesions [12]. Histopathology with hematoxylin-eosin staining is highly specific but may miss characteristic intranuclear inclusions due to their patchy distribution. IHC improves diagnostic accuracy by detecting CMV antigens within infected cells and is considered the most reliable method when PCR testing is unavailable [9,13].

A study by Roblin et al. demonstrated that CMV DNA is almost exclusively detected in inflamed mucosal areas, emphasizing the importance of targeted biopsies from ulcer bases and margins when CMV colitis is suspected [13].

Despite increasing awareness, the true prevalence of CMV colitis among IBD patients remains variable, largely due to heterogeneous testing strategies across institutions. The updated European Crohn's and Colitis Organisation (ECCO) guidelines recommend CMV screening in severe or steroid-refractory IBD and before initiating immunosuppressive therapy.

The aim of this study was to determine the frequency of acute CMV infection and CMV colitis among hospitalized IBD patients in a tertiary care center and to describe associated clinical, endoscopic, and pathological features. We evaluated CMV IgG and immunoglobulin M (IgM) serology and performed colonoscopy with targeted biopsies and IHC when indicated.

## Materials and methods

Study design

This retrospective descriptive study was conducted in the Gastroenterology Clinic of the Emergency Clinical County Hospital of Târgu Mureș, Romania, between April 1, 2024, and December 31, 2024.

Study population and sample size

The electronic database of the clinic and the medical records of hospitalized patients were reviewed to identify all individuals with a confirmed diagnosis of IBD who had undergone CMV serology and/or colonoscopy with mucosal biopsies. All patients who met these criteria were evaluated for inclusion. Eligible participants were adults aged 18 years or older, with an established diagnosis of either CD or UC. Patients were excluded if clinical or laboratory data were incomplete. Patients with indeterminate colitis were also excluded. The final sample consisted of 54 patients.

Study measures

CMV colitis was defined as the presence of endoscopically visible colonic inflammation, manifesting as ulcers, erosions, or severe mucosal changes, accompanied by the histopathological confirmation of CMV infection, either through the identification of characteristic "owl's eye" intranuclear inclusions on hematoxylin-eosin staining or through positive IHC for CMV antigens. Active IBD was defined using the Mayo score for UC and Crohn's Disease Activity Index (CDAI) for CD. 

Demographic information (age, sex), disease characteristics (type of IBD and disease duration), and therapeutic history (use of corticosteroids, biologics, immunomodulators, or 5-aminosalicylic acids) were extracted from the institutional IBD database and medical charts. Laboratory parameters were obtained from the Central Laboratory of the Emergency Clinical County Hospital of Târgu Mureș, using the following reference intervals: fecal calprotectin 0-50 µg/g, hemoglobin 12-15 g/dL, iron 12-28 µmol/L, albumin 3.97-4.94 g/dL, aspartate aminotransferase (AST) 5-34 U/L, alanine aminotransferase (ALT) 0-33 U/L, erythrocyte sedimentation rate (ESR) 1-12 mm/h, CMV IgG 0-0.49 U/mL, and CMV IgM 0-0.7 index CO. C-reactive protein (CRP) was evaluated qualitatively.

CMV testing was performed at the discretion of the treating physician, particularly in the setting of active disease, steroid-refractory or steroid-dependent course, or severe endoscopic findings. In patients in clinical remission, evaluation was prompted by persistent inflammatory markers and/or mucosal lesions.

All enrolled patients underwent full colonoscopy performed by experienced gastroenterologists, with multiple biopsies obtained from areas showing endoscopic abnormalities such as erythema, edema, friability, ulcers, or aphthoid lesions suggestive of active IBD. All biopsy specimens were analyzed by the same gastrointestinal pathologist following a standardized diagnostic protocol, including routine histopathology and immunohistochemical staining when CMV infection was suspected clinically or endoscopically.

Statistical analysis

Statistical analysis was primarily descriptive. Nominal variables were summarized as absolute numbers and percentages. Continuous variables were expressed as means ± standard deviation or medians (interquartile range), as appropriate. Formal inferential statistical analyses regarding CMV status were not performed. Statistical analyses were conducted using Microsoft Excel (Microsoft Corp., Redmond, WA, USA) and the GraphPad software (Insight Venture Management, LLC, New York, NY, USA).

Ethics statement

The study adhered to the principles outlined in the Declaration of Helsinki and the applicable Romanian legislation governing biomedical research. Ethical approval was obtained from the Ethics Committee of the Emergency Clinical County Hospital of Târgu Mureș (approval number: 5960/15.03.2024).

## Results

**Figure 1:**
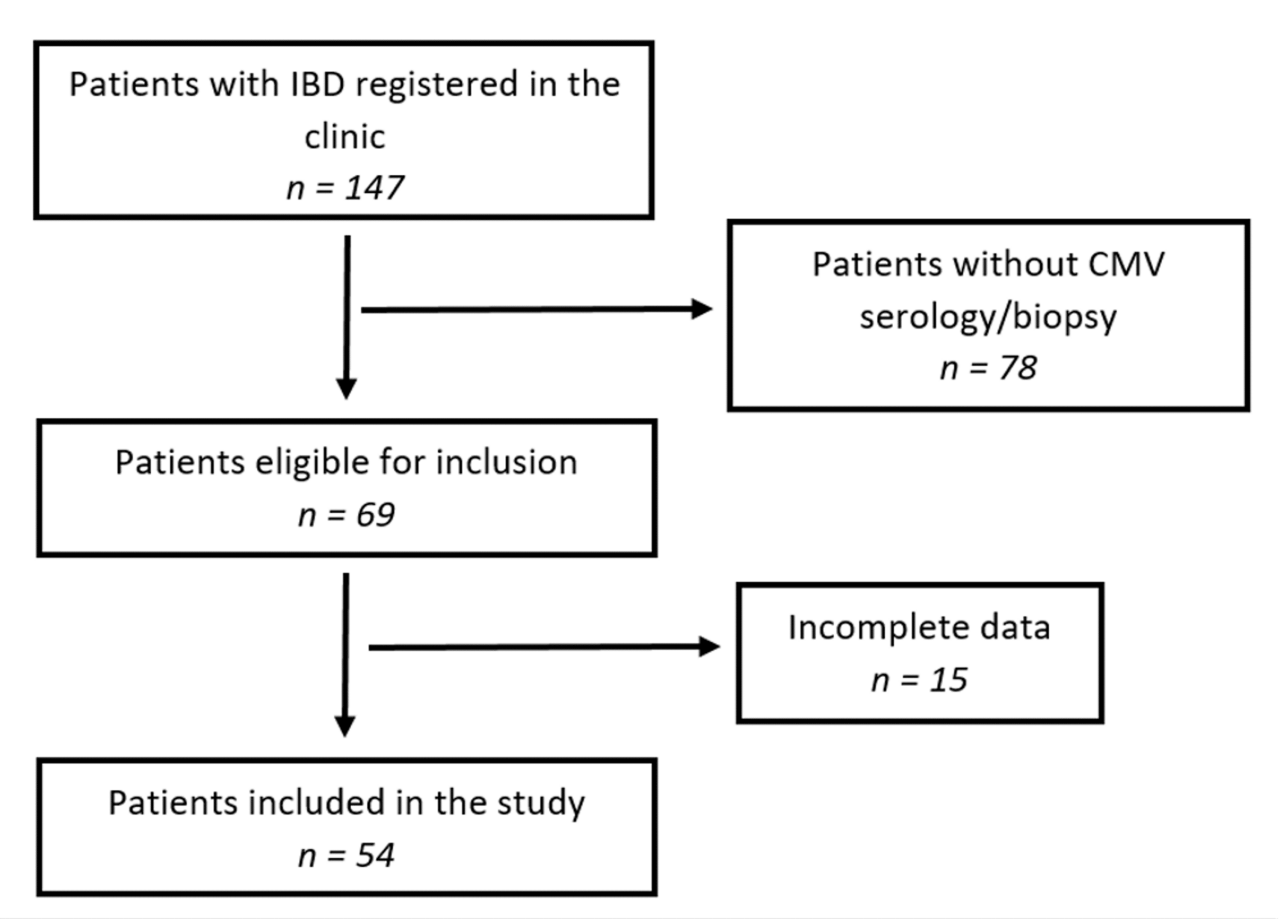
Patient selection flowchart. IBD: inflammatory bowel disease; CMV: cytomegalovirus

Between April 1, 2024, and December 31, 2024, a total of 54 patients with IBD were evaluated in the Gastroenterology Clinic. Figure 1 shows the flowchart of the patient selection. The mean age of the study population was 41 years, ranging from 18 to 86 years. Most patients (37 cases (68.51%)) were hospitalized for routine follow-up, while 17 patients (31.48%) presented with symptoms suggestive of active disease, including abdominal pain, diarrhea, bloody stools, and weight loss.

A predominance of male patients was observed, representing 61.54% (n=32) of the cohort. The single patient diagnosed with CMV colitis was male, as were two patients who tested positive for CMV IgM. Serologic evaluation showed that three patients (5.55%) tested negative for both CMV IgG and IgM and two patients (3.70%) were IgG-negative but IgM-positive, while the remaining 49 patients (90.74%) were IgG-positive and IgM-negative. In Table 1, the demographic characteristics and types of investigations performed are presented.

**Table 1:** Demographic characteristics and types of investigations performed.

Variable	Category	n (%)
Type of disease	Ulcerative colitis	30 (55.56%)
	Proctitis	1 (3.33%)
	Left-sided colitis	13 (43.33%)
	Pancolitis	16 (53.33%)
	Crohn's disease	24 (44.44%)
	Ileitis	1 (4.17%)
	Ileocolitis	9 (37.50%)
	Crohn's colitis	14 (58.33%)
Sex	Female	22 (40.74%)
	Male	32 (59.26%)
Age groups	18-30 years	16 (29.63%)
	31-50 years	22 (40.74%)
	51-70 years	13 (24.07%)
	>70 years	3 (5.56%)
Type of investigation	Serology only	12 (22.22%)
	Biopsy only	12 (22.22%)
	Serology+biopsy	30 (55.56%)
Immunohistochemistry	Yes	15 (35.71%)
	No	27 (64.29%)

Regarding disease duration, the average time since IBD diagnosis was 6.14 years, with values ranging from three months to a maximum of 26 years. Of the total study cohort, 30 patients (55.56%) had UC, and 24 patients (44.44%) had CD. The only patient diagnosed with acute CMV colitis belonged to the CD group.

Among patients with UC, disease localization included proctitis in one patient (3.33%), left-sided colitis in 13 patients (43.33%), and pancolitis in 16 patients (53.33%). In the CD group, one patient (4.17%) had ileitis, nine patients (37.50%) had ileocolitis, and 14 patients (58.33%) had colonic involvement. Regarding disease activity, 37 patients (68.51%) were in clinical remission, while 17 patients (31.48%) presented with active disease at the time of evaluation. Based on the Mayo score, 10 patients (33%) with UC had active disease (four with mild disease, four with moderate disease, and two with severe disease). According to the CDAI, seven patients (29%) with CD had active disease (two with mild disease, four with moderate disease, and one with severe disease).

Of the patients with positive CMV IgM or biopsy findings, clinical manifestations varied. The patient with confirmed CMV colitis reported diarrhea, bloody stools, fatigue, and an approximate 3-kg weight loss. In contrast, the two patients with positive CMV IgM serology but negative biopsy results were hospitalized for routine assessment and did not present symptoms of active colitis.

Antiviral therapy consisting of intravenous ganciclovir 5 mg/kg for five days followed by oral valganciclovir 900 mg twice a day for 10 days was administered to the patient with histologically confirmed CMV colitis. Follow-up data for this patient were not available, as the patient did not attend scheduled post-treatment visits, preventing further assessment of clinical outcomes.

With regard to diagnostic methods, combined serology and biopsy were performed in 30 patients (55.56%), while serology alone was obtained in 12 patients (22.22%). Biopsy without accompanying serology was performed in an additional 12 patients (22.22%). Among all patients who underwent biopsy, IHC staining for CMV was performed in 15 cases (35.71%). Only one patient (1.85%) was diagnosed with CMV colitis based on biopsy and IHC results. Two patients (3.70%) showed elevated CMV IgM levels with negative IgG and normal biopsy findings.

Assessment of medical therapy revealed that, at the time of CMV testing, 29 patients (53.70%) were receiving biologic therapy, 18 patients (33.33%) were treated with 5-aminosalicylic acid, four patients (7.41%) were on corticosteroids, and three patients (5.56%) were treated with azathioprine (Table 2). Among the 29 patients on biologics, six patients (20.69%; two with CD and four with UC) required a second-line biologic agent due to loss of response or treatment failure. The most common treatment escalation pattern was from adalimumab to vedolizumab. One patient required a third-line biologic sequence consisting of adalimumab followed by vedolizumab and then ustekinumab. The mean duration of first-line biologic therapy was 41 months for adalimumab and 21 months for vedolizumab. The average duration of second-line vedolizumab therapy was 15.8 months. All three patients with evidence of CMV infection had received corticosteroid therapy.

**Table 2:** Treatment patterns and their distribution among IBD patients. IBD: inflammatory bowel disease; CMV: cytomegalovirus

Treatment type	n (%)	CMV colitis (n)
Biologic therapy	29 (53.70%)	0
Adalimumab	20 (68.97%)	0
Infliximab	2 (6.90%)	0
Vedolizumab	6 (20.69%)	0
Ustekinumab	1 (3.45%)	0
5-Aminosalicylic acid	18 (33.33%)	0
Corticosteroids	4 (7.41%)	1
Azathioprine	3 (5.56%)	0

Laboratory evaluation revealed elevated fecal calprotectin in 19 patients (35.19%). Anemia was present in 15 patients (27.78%), hyposideremia in 10 patients (18.52%), and hypoalbuminemia in six patients (11.11%). Elevated liver enzymes were detected in three patients (5.56%). Inflammatory markers were frequently abnormal, with elevated CRP in 17 patients (31.48%) and elevated ESR in 24 patients (44.44%). Laboratory abnormalities identified in the study cohort are summarized in Table 3.

**Table 3:** Distribution of laboratory abnormalities in the study cohort. CRP: C-reactive protein; ESR: erythrocyte sedimentation rate

Parameter	Category	n (%)
Fecal calprotectin	Normal	35 (64.81%)
	Elevated	19 (35.19%)
Hemoglobin	Normal	39 (72.22%)
	Anemia	15 (27.78%)
Iron	Normal	44 (81.48%)
	Hyposideremia	10 (18.52%)
Albumin	Normal	48 (88.89%)
	Hypoalbuminemia	6 (11.11%)
Liver enzymes	Normal	51 (94.44%)
	Elevated	3 (5.56%)
CRP	Normal	37 (68.52%)
	Elevated	17 (31.48%)
ESR	Normal	30 (55.56%)
	Elevated	24 (44.44%)

## Discussion

Diagnosing CMV colitis in patients with IBD remains a significant clinical challenge, as symptoms and endoscopic findings frequently overlap with those of severe disease flares. Updated ECCO guidelines recommend screening for CMV in all patients with moderate-to-severe or steroid-refractory IBD, particularly before initiating immunosuppressive therapy [14,15]. CMV colitis has been associated with adverse clinical outcomes, including severe flare-ups, steroid-refractory disease, fulminant colitis, and the need for colectomy. In our study, only one patient (1.85%), a case of CD, was diagnosed with CMV colitis based on histopathology and IHC. This reflects a low frequency among patients who underwent CMV evaluation, comparable to rates reported in similar selectively tested cohorts.

IHC remains the gold standard for diagnosing CMV colitis due to its superior sensitivity and specificity compared to routine hematoxylin-eosin staining [9]. In our cohort, three patients showed serologic evidence of acute infection, as indicated by positive CMV IgM; however, only one had the histopathologic confirmation of CMV colitis. The remaining two patients were IgM-positive but had negative biopsy findings. These results reiterate the limited utility of serologic testing alone in diagnosing active CMV colitis, which is consistent with existing literature. Pediatric guidelines similarly emphasize that CMV detection in peripheral blood is not clinically meaningful in acute severe UC, further supporting the limited diagnostic value of serology in the absence of mucosal tissue evaluation [16,17]. Multiple studies confirm that serology primarily identifies prior exposure rather than active infection and is therefore insufficient as a standalone diagnostic tool [6].

Four patients in our study had received corticosteroids at the time of CMV testing, and among them, one was diagnosed with CMV colitis and two were IgM-positive. These observations may be consistent with previous evidence suggesting that corticosteroid therapy is associated with CMV reactivation in immunocompromised hosts. However, given the very limited number of cases, this finding should be interpreted as hypothesis-generating.

We also assessed therapeutic patterns in the study population. More than half of the patients (53.7%) were receiving biologic therapy at the time of CMV testing, and none of them were diagnosed with CMV colitis in this small cohort. Existing research has demonstrated that infliximab therapy is not associated with an increased risk of CMV reactivation in IBD [18]. Lavagna et al. reported a CMV seroprevalence of 70% among patients with active CD initiating infliximab therapy, with all patients remaining negative for CMV DNA on blood PCR after 14 weeks of follow-up [19]. In our cohort, the most frequent treatment sequence involved initial therapy with adalimumab followed by vedolizumab in cases of inadequate response, reflecting earlier clinical guidelines and local practice. However, recent evidence suggests that initiating treatment with a non-anti-TNF agent such as vedolizumab may be beneficial in selected patient populations with CD or UC [20].

This study has several limitations. One of the major limitations is the relatively small sample size, which restricts the ability to draw robust conclusions or perform meaningful statistical analyses, particularly regarding associations between CMV status and clinical variables. Importantly, the majority of included patients were hospitalized for routine follow-up and were in clinical remission, while only a small proportion had active or steroid-refractory disease. This resulted in a significant selection bias toward a lower-risk population, thereby limiting the generalizability of our findings and likely leading to an underestimation of the true prevalence of CMV colitis in hospitalized IBD patients.

Additionally, corticosteroid use was infrequent, further reducing the likelihood of detecting CMV reactivation in this cohort. Another major limitation is the unavailability of CMV PCR testing, which would have strengthened diagnostic accuracy and provided information on systemic viral replication. The lack of standardized disease activity indices and endoscopic severity scores represents an additional limitation, as these parameters are crucial in characterizing disease severity and correlating CMV status with clinical outcomes. Moreover, data on comorbidities, BMI, and concomitant medications were not consistently recorded, preventing the assessment of potential confounding factors that may influence CMV reactivation risk.

An additional limitation is the lack of a standardized diagnostic protocol for CMV infection across the cohort. IHC was performed only in a subset of patients, CMV PCR testing was unavailable, and colonic biopsies were obtained primarily when CMV infection was clinically suspected. This non-uniform diagnostic approach may have resulted in detection bias and the underdiagnosis of CMV colitis, potentially leading to an underestimation of its true prevalence in this population.

We also acknowledge limitations related to biologic therapy data. Although we reported the types of biologic agents used, detailed information regarding dosing intervals, cumulative exposure, reasons for switching therapy, and treatment duration was incomplete. These missing details limited our ability to explore potential relationships between biologic regimens and CMV infection or reactivation.

Due to the extremely low event rate and lack of standardized diagnostic and severity assessment protocols, our study was not designed to identify risk factors or establish causal associations. Any observed relationships, particularly regarding corticosteroid exposure, should therefore be interpreted with caution.

Despite these limitations, our study provides relevant information on the frequency and clinical patterns of CMV infection among hospitalized IBD patients in a Romanian tertiary care center. The findings reinforce the importance of targeted mucosal biopsy and IHC in appropriately selected patients and support the recommendation that CMV should be considered in cases of severe or steroid-refractory IBD. Future research involving larger multicenter cohorts and standardized diagnostic protocols, including PCR testing and validated disease activity indices, is warranted to better define the epidemiology and clinical impact of CMV in IBD.

## Conclusions

The frequency of CMV colitis among hospitalized IBD patients who underwent CMV evaluation in our cohort was low. Nevertheless, its identification underscores the importance of maintaining clinical vigilance, particularly in individuals receiving immunosuppressive therapy. The diagnosis of CMV colitis primarily relies on targeted mucosal biopsies and IHC, with serology playing only a limited adjunctive role. Although our findings raise the hypothesis of a possible association between corticosteroid exposure and CMV infection, this observation should be interpreted cautiously due to the very small number of cases. Larger, multicenter studies incorporating standardized diagnostic procedures are needed to better define the epidemiology and clinical impact of CMV colitis in the IBD population.
